# Successful Outcome of Modified Quad Surgical Procedure in Preteen and Teen Patients with Brachial Plexus Birth Palsy

**Published:** 2012-12-04

**Authors:** Rahul K. Nath, Chandra Somasundaram

**Affiliations:** Texas Nerve and Paralysis Institute, Houston

## Abstract

**Objective:** To evaluate the outcome of modified Quad procedure in preteen and teen patients with brachial plexus birth palsy. **Background:** We have previously demonstrated a significant improvement in shoulder abduction, resulting from the modified Quad procedure in children (mean age 2.5 years; range, 0.5–9 years) with obstetric brachial plexus injury. **Methods:** We describe in this report the outcome of 16 patients (6 girls and 10 boys; 7 preteen and 9 teen) who have undergone the modified Quad procedure for the correction of the shoulder function, specifically abduction. The patients underwent transfer of the latissimus dorsi and teres major muscles, release of contractures of subscapularis pectoralis major and minor, and axillary nerve decompression and neurolysis (the modified Quad procedure). Mean age of these patients at surgery was 13.5 years (range, 10.1–17.9 years). **Results:** The mean preoperative total Mallet score was 14.8 (range, 10–20), and active abduction was 84° (range, 20°–140°). At a mean follow-up of 1.5 years, the mean postoperative total Mallet score increased to 19.7 (range, 13–25, *P* < .0001), and the mean active abduction improved to 132° (range, 40°–180°, *P* < .0003). **Conclusion:** The modified Quad procedure greatly improves not only the active abduction but also other shoulder functions in preteen and teen patients, as this outcome is the combined result of decompression and neurolysis of the axillary nerve and the release of the contracted internal rotators of the shoulder.

The most common obstetric brachial plexus injury (OBPI) involves lesions to upper trunk (C5-C6), with or without injury to the C7 nerve root. C8-T1 roots are rarely affected. The injury can be simple stretch or rupture or avulsion. The incidence of OBPI is about 0.15% in United States.^1^ Most of these injuries are transient; patients recover functions spontaneously within the 3 months of life. However, some result in prolonged and persistent disability even after therapy and therefore they are considered for surgical treatments.

We have previously reported a significant improvement in abduction resulting from the modified Quad surgical procedure in OBPI patients.^2^ However, we did not find a significant correlation between the abduction resulting from mod Quad and the age of the OBPI patients. Our published study patient population age was only up to 9 years.^2^ Therefore, we further analyzed whether there is any significant difference in the functional outcome of modified Quad procedure in various age groups, especially in preteen and teen patients, who have not undergone any other surgical procedures for this injury. We found significant improvement of the Mallet functions after at least a year follow-up of modified Quad surgery in preteen and teenage patients. In addition, there was no significant correlation between the age of these patients and the improvement in Mallet functions in the present study.

## PATIENTS AND METHODS

Sixteen patients (10 boys and 6 girls; 7 preteen and 9 teen) with obstetric brachial plexus palsy that had not fully recovered by the age of 6 months. Of these, 9 patients had a C5-C6 injury, 3 a C5-C7 injury, 2 a C5-C8, and 2 a C5-T1 injury. The patients were operated on between 2006 and 2011. The mean age of these patients at surgery was 13.5 years (range, 10.1–17.9 years). No patients with complete follow-up were excluded. The patients in this study showed significant abduction deformity with contractures of the latissimus dorsi, the teres major, and the pectoralis major muscles. Modified Quad surgical procedure was performed on these patients as we have reported previously.^2^ Statistical analysis was performed using Analyse-it plugin (Leeds, England) for Microsoft Excel 2003 software. A *P* value of less than .05 was considered as statistically significant.

## RESULTS

**Figure 1:**
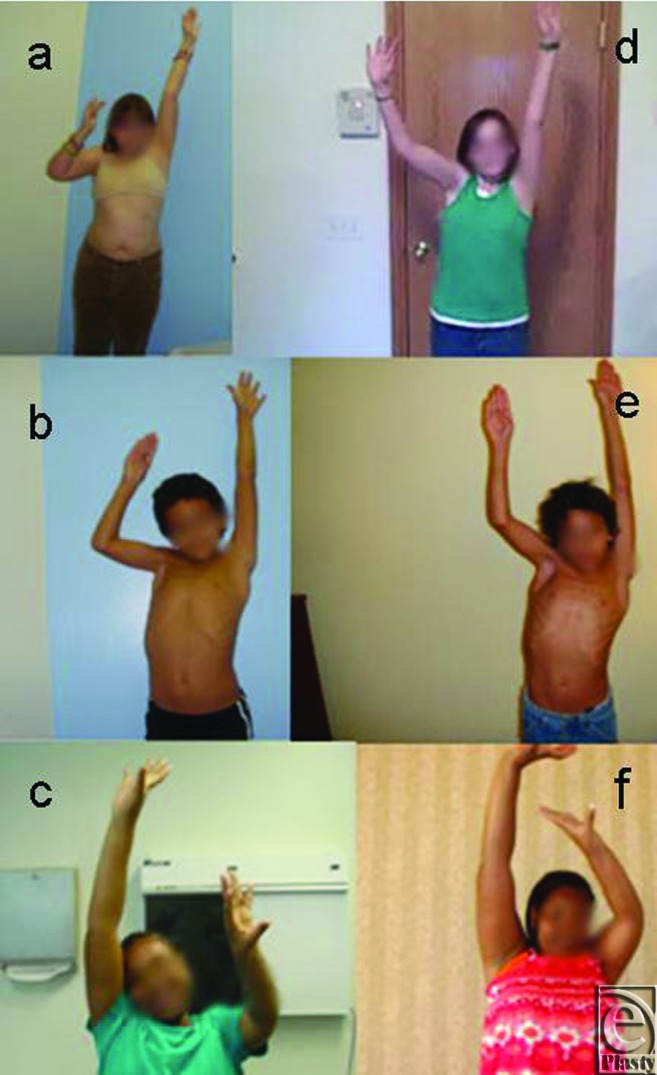
Comparisons of preoperative (a–c) and postoperative (d–f) shoulder abduction in preteen and teen patients with obstetric brachial plexus injury.

**Figure 2:**
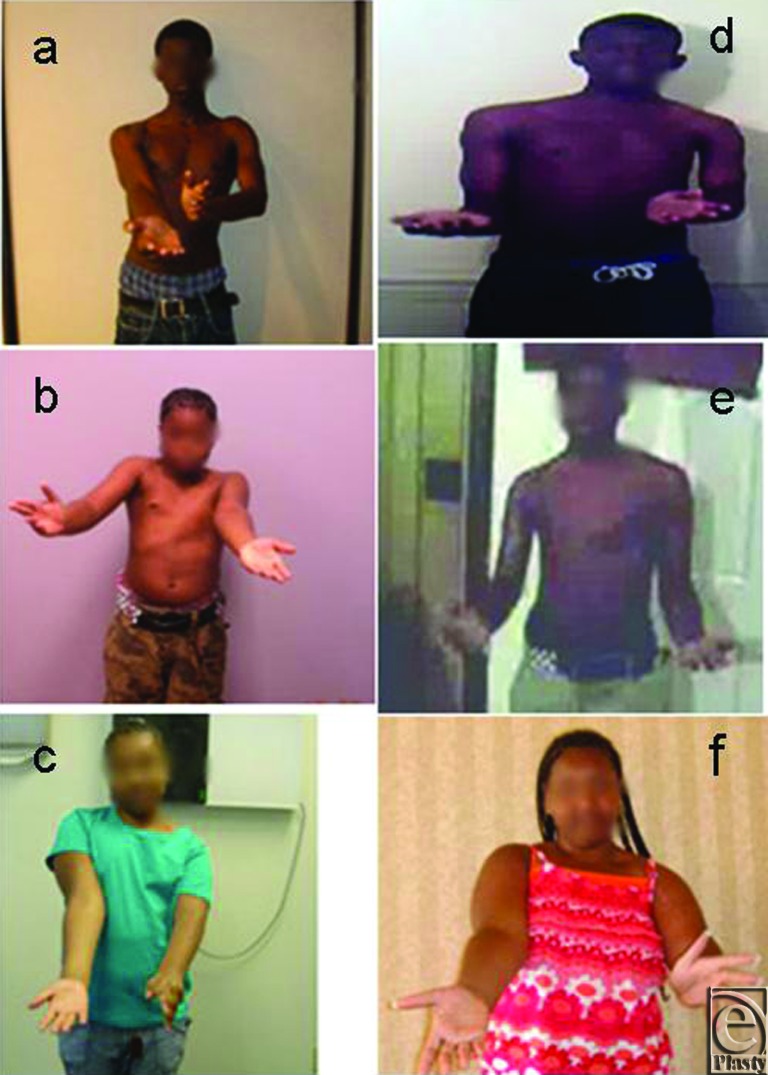
Comparisons of preoperative (a–c) and postoperative (d–f) supination in preteen and teen patients with obstetric brachial plexus injury.

There was a significant improvement in abduction and all Mallet functions in these patients after at least 1-year follow-up (Table 1). The mean preoperative total Mallet score was 14.8 (range, 10–20), and active abduction was 83° (range, 20°–140°). At a follow-up of 1.5 years, the mean postoperative total Mallet score increased to 19.7 (range, 13–25, *P* < .0001) and the mean active abduction improved to 132° (range, 50°–180°, *P* < .0003) (Table 1 and Fig 1). There was no correlation between the outcome in terms of total Mallet score and the age of these patients. However, the improvement in abduction was directly correlated to the age of these patients. We also achieved significantly higher external rotation postoperatively (mean postoperative Mallet score, 3.9; *P* < .0001), which was improved from preoperative Mallet score of 2.8. Supination angle improved to 32 (*P* < .007), which was preoperatively at −1.9 (Table 1). Modified Mallet functional movements and supination were evaluated for each patient and reported (Table 1 and Fig 2).

## DISCUSSION

The patients in our present study are all preteen and teen who have suffered brachial plexus injury at birth. They have not had any other surgical procedures at our institution. However, 2 patients had nerve transfer/graft and 5 patients had muscle or tendon transfer procedures in other institutions. Yet, 5 of these 7 patients had severe contractures (20°–40° abduction) on presentation at our institution. Although many surgical procedures have been described, muscle releases and tendon transfers are traditionally used to treat this deformity. We recently demonstrated that OBPI patients who underwent nerve reconstructions developed more serious shoulder complications and therefore required more secondary surgical procedures to address this deformity.^3^ The weakness of the deltoid, infraspinatus and supraspinatus, and teres minor in combination with the strength of the relatively spared large internal rotator and adductor muscles causes progressive muscle imbalance, resulting in loss of abduction range and external rotation. All the patients in our present study underwent surgical contracture releases and tendon transfers as described in the modified Quad protocol in methods.

Compression of the axillary nerve in the quadrangular space is a common cause for impairment in these patients. Therefore, we performed decompression of the axillary nerve and released the long head of the triceps, which address the periarticular tightness in these patients. Latissimus dorsi and teres major transfers to the teres minor enhance the stabilizing effect of the rotator cuff. This enables the deltoid to act more effectively. There was great improvement in all Mallet functions in addition to abduction, especially external rotation and supination (Table 1 and Fig 2), at least a year after the modified Quad surgery. Other investigators^4^^-^10 have demonstrated improvement in external rotation by performing the tendon transfers higher and more laterally in the shoulder than those performed in our study. Although these published surgical techniques have improved external rotation, yet they have inhibited the abduction and flexion achievable.^4^^-^10 Our modified Quad procedure considers not only abduction but also other shoulder functions, specifically external rotation and supination. We followed the axillary nerve decompression as described by Adelson et al^11^ and kept the muscle transfers as low as possible. After the procedure, it was easy for the weakened abductors to perform abduction without the tethering effect of the stronger muscles, which were released. Although these patients are adolescents and teen, 11 of the 17 (65%) patients had active abduction of 140° and more. We achieved good results even in patients with very poor preoperative function. Some of our patients had very adverse preoperative abduction values (<20°). Our results are novel and appear superior to other published studies, as these outcomes are in teen and preteen (adolescent) patients.

## CONCLUSION

Release of the contracted adductor muscles of the shoulder and tendon transfers along with decompression and neurolysis of the axillary nerve greatly improve active abduction and other shoulder functions not only in young pediatric patients (0.5–9.0 years old) but also in preteen and teen patients (adolescents) with muscle imbalance secondary to brachial plexus injury sustained at birth.

## Figures and Tables

**Table 1:** Outcome of modified Quad surgery in preteen and teen patients with obstetric brachial plexus injury (N = 16)*

	Abduction,† °	External rotation†	Hand to neck†	Hand to spine†	Hand to mouth†	Supination§	Total Mallet score†
Preoperative	3.3 ± 0.9 (84 ± 50)	2.8 ± 0.8	2.9 ± 1.0	2.6 ± 0.7	3.3 ± 1.0	2.8 ± 1.1	14.8 ± 3.5
Postoperative Mean follow-up 1.5 y	4.2 ± 0.8 (134 ± 49)	3.9 ± 0.9	4.0 ± 1.1	3.2 ± 1.1	4.3 ± 0.5	3.6 ± 1.2	19.7 ± 3.4

*Comparison of pre- and postoperative modified Mallet score.

†*P* < .0001.

‡*P* < .03.

§*P* < .007.
